# A scoping review of the distribution and frequency of extended-spectrum *β*-lactamase (ESBL)-producing Enterobacteriaceae in shrimp and salmon

**DOI:** 10.1017/S0950268822001819

**Published:** 2022-12-05

**Authors:** K. M. Young, M. J. Isada, M. Reist, F. C. Uhland, L. M. Sherk, C. A. Carson

**Affiliations:** 1 Public Health Risk Sciences Division, National Microbiology Laboratory, Public Health Agency of Canada, Guelph, Ontario, Canada; 2 Centre for Foodborne, Environmental and Zoonotic Infectious Diseases, Public Health Agency of Canada, Guelph, Ontario, Canada; 3 Veterinary Drugs Directorate, Health Products and Food Branch, Health Canada, Ottawa, Ontario, Canada

**Keywords:** Enterobacteriaceae, food safety, scoping review, seafood, antimicrobial resistance

## Abstract

Antimicrobial-resistant (AMR) bacteria are a threat to public health as they can resist treatment and pass along genetic material that allows other bacteria to become drug-resistant. To assess foodborne AMR risk, the Codex Guidelines for Risk Analysis of Foodborne AMR provide a framework for risk profiles and risk assessments. Several elements of a risk profile may benefit from a scoping review (ScR). To contribute to a larger risk profile structured according to the Codex Guidelines, our objective was to conduct a ScR of the current state of knowledge on the distribution, frequency and concentrations of extended-spectrum *β*-lactamase (ESBL)-producing Enterobacteriaceae in salmon and shrimp. Articles were identified via a comprehensive search of five bibliographic databases. Two reviewers screened titles and abstracts for relevance and characterised full-text articles with screening forms developed *a priori*. Sixteen relevant studies were identified. This review found that there is a lack of Canadian data regarding ESBL-producing Enterobacteriaceae in salmon and shrimp. However, ESBL- producing *Escherichia coli*, *Klebsiella pneumoniae* and other *Enterobacteriaceae* have been isolated in multiple regions with a history of exporting seafood to Canada. The literature described herein will support future decision-making on this issue as research/surveillance and subsequent assessments are currently lacking.

## Introduction

Antimicrobial-resistant (AMR) bacteria are a growing issue of global concern, threatening our capacity to treat common infections, prolonging the duration of hospitalisations and increasing treatment costs and mortality among infected patients [1, 2]. Antimicrobial use (AMU) in the community, healthcare and agriculture sectors are thought to be the principle drivers of increases in antimicrobial resistance (AMR) [3]. The recruitment of resistance genes in the environmental resistome, genetic mutations and inter or intra-species exchange of antimicrobial resistance genes (ARGs) between bacteria are also of recognised importance [4].

The Codex Guidelines for Risk Analysis of Foodborne AMR [5] provide a useful and transparent way to assess the risk of AMR arising through the food chain. One component of the risk analyses pathway is a risk profile; suggested elements for inclusion are described in detail in the Codex Guidelines [5]. A foodborne AMR risk profile presents the current state of scientific information on identified AMR food safety issues to inform risk managers prior to decision-making. A risk profile incorporates various elements for consideration including information on antimicrobial-resistant (AMR) microorganism(s) and/or determinant(s). One of the challenges with conducting risk profiles, is providing pertinent information in a timely manner. A scoping review (ScR) is an ideal synthesis tool to use when the aim is to identify and map available evidence on a given topic [6, 7]. Several suggested elements within a risk profile lend themselves to ScRs while other elements need only succinct targeted pieces of information to address risk managers questions. In this case, we were interested in describing the findings and range of research related to the distribution, frequency and concentrations of extended-spectrum *β*-lactamase (ESBL)- producing bacteria in salmon and shrimp.

In terms of the AMR hazard, in 2017, the World Health Organization (WHO) published a list of antibiotic-resistant ‘priority pathogens’ that pose the greatest threat to human health [8]. Priority 1 is considered ‘critical’ and includes extended-spectrum *β*-lactamase-producing Enterobacteriaceae (ESBL-E) [8]. These bacteria can cause severe infections and have become resistant to many antimicrobials, including the best available treatments for multi-drug resistant bacteria [8]. ESBL-E are a group of organisms which demonstrate resistance to 3rd generation cephalosporins such as cefuroxime, cefotaxime, ceftriaxone, ceftazidime, cefepime and cefpirome [9, 10]. They are also able to hydrolyse a wide range of *β*-lactams, including numerous penicillins and monobactams. ESBLs are mainly encoded on plasmids but may also be present in the chromosome [9]. ESBLs consist of class A *β*-lactamases including TEM/SHV derivatives and CTX-M enzymes (e.g., CTX-M-1, CTX-M-15 and CTX-M-55, CTX-M-2, CTX-M-8, CTX-M-9 and CTX-M-25) [11]. There are 183 and 178 variants of TEM and SHV enzymes, respectively; however, not all of them are ESBLs [12].

Resistance to 3^rd^ generation cephalosporins is alarming, as they are considered high priority antimicrobials by the WHO, of ‘very high importance’ in human medicine according to Health Canada's categorisation, and are used as ‘last resort’ treatments in some multidrug-resistant infections [13, 14]. Resistance to 3rd generation cephalosporins can correlate with the presence of ESBL genes (*bla*_TEM_, *bla*_SHV_ and *bla*_CTX−M_) [15]. Of particular note, bacteria containing ESBL genes may exhibit high levels of cross-resistance to various human and veterinary cephalosporin formulations (ceftriaxone, ceftazidime, cofotaxime, ceftiofur) [16].

Enterobacteriaceae encompass several important human pathogens including *Klebsiella pneumonia*, *Escherichia coli*, *Enterobacter* spp., *Serratia* spp., *Proteus* spp., *Providencia* spp and *Morganella* spp, which have been reported in agriculture and aquatic animal production systems [17, 18]. Of particular relevance, their ubiquitous presence and propensity for genetic exchange make them potential reservoirs of AMR genes [19].

In Canada, there has been a recent discovery of Enterobacteriaceae that possess and express carbapenemase and *β*-lactamase–conferring genes in isolates from imported retail seafood [20]. Salmon and shrimp are of interest because they are the most commonly consumed seafood products in Canada [21]. Most salmon and shrimp products consumed in Canada are farmed (aquacultured). Atlantic salmon (*Salmo salar*) is the main farmed salmon species, is primarily produced domestically, and its production is subject to strict regulations on site selection, quality control and AMU [22, 23]. The main farmed shrimp species worldwide is the white legged shrimp (*Litopenaeus vannamei*) [24]. Shrimp are mostly grown and imported to Canada from Central America and Southeastern Asia [23], where several classes of antimicrobials are used and regulations concerning use and enforcement are different from those in Canada [25].

The objective of this study was to conduct a ScR of the current state of knowledge on the distribution, frequency and concentrations of ESBL-E in salmon and shrimp. Information from this ScR will contribute to a larger risk profile of ESBL-producing bacteria from seafood following the Codex guidelines. To our knowledge, this is the only ScR of ESBL-E in these popular seafood commodities. A secondary objective was to identify areas where more research is needed and support evidence-informed decision-making concerning the need for ongoing surveillance of AMR in seafood in Canada.

## Methods

This ScR was designed to answer the following question: ‘What is the distribution, frequency and/or concentrations of extended-spectrum *β*-lactamase (ESBL)- producing Enterobacteriaceae in shrimp and salmon?’. At the time this review was initiated (2019), the bacterial scientific order was labelled as ‘Enterobacteriaceae’, however, in 2020 a taxonomy change was implemented to make the change to ‘Enterobacterales’ [26]. For consistency between the *a priori* study protocol and results we have decided to use the terminology ‘Enterobacteriaceae’ throughout this review. A ScR protocol was created *a priori* to ensure reproducible, transparent and consistent results. The protocol includes a rationale for the project, search algorithms, abstract screening form and data characterisation form. The protocol can be found in the Supplementary Material. Creation of the protocol and execution of the review was completed by a team of individuals with multi-disciplinary expertise in epidemiology, public health, knowledge synthesis, risk assessment, aquaculture and veterinary medicine. This ScR followed the Preferred Reporting Items for Systematic review and Meta-Analyses extension for Scoping Reviews (PRISMA-ScR) guidelines [27].

### Search strategy

The full electronic search strategy can be found in the ScR protocol (Supplemental Material). In brief, the search strategy included general AMR terms (e.g., ‘Drug Resistance’), ESBL specific terms (e.g., ‘ESBL’ or ‘beta-lactams’), population terms (e.g., ‘salmon’ or ‘shrimp’ or ‘seafood’) and bacteria terms (e.g., ‘*E. coli*’ or ‘*Salmonella*’ or ‘Enterobacteriaceae’) combined into search strings/algorithms. There was no limit on publication date.

The initial search was conducted in November 2019 and an update was conducted in February 2021. These searches were conducted in five bibliographic databases: MEDLINE® (1946 to current), AGRICOLA™ (1970 to current), EMBASE® (1974 to current), CAB Abstracts (1973 to current) and Food Science and Technology Abstracts (1969 to current). The electronic search was verified by hand searching the reference lists of three literature reviews focused or partially focused on AMR in salmon or shrimp [28–30]. Spot checks were also done in reference lists of other relevant literature as the authors were reviewing.

A search was also conducted in Google Scholar using the algorithm: (antibiotic resistance) and (ESBL) and (Enterobacteriaceae) and (salmon or shrimp). To limit the amount of irrelevant articles in our search, only articles that mentioned AMR were considered for inclusion.

### Relevance screening and inclusion criteria

A relevance screening form was developed *a priori*. Abstracts, titles and keywords were screened for relevance using the following question: Does this citation describe primary research on: presence of ESBLs in Enterobacteriaceae of salmon and shrimp? Two reviewers screened titles and abstracts for relevance and any conflicts were resolved by a third reviewer.

### Study characterisation and extraction

The study characterisation form was developed *a priori* and can be found in the ScR protocol (Supplemental Material). The study characterisation form aimed to classify and characterise the research on ESBL-E from salmon and shrimp. The form first reconfirmed the relevance of the publication prior to extracting important characteristics of the study. This included the following study details: language (due to limited translation resources, articles published in languages other than English or French were excluded), location, study date, document type, focus of paper (i.e., relevant to ESBLs in the food chain), food commodity, if AMR data were extractable for each food commodity, Enterobacteriaceae investigated, and outcomes. Two reviewers characterised full-text articles and any conflicts were resolved by a third reviewer.

### Scoping review management, data charting and analysis

The search strategy results were exported and de-duplicated in Mendeley (Mendeley Ltd, 2021). These articles were then exported to Rayyan, a web-based systematic review software, where relevance screening (keyword and abstract only) was conducted [31]. Full text of articles deemed relevant were then procured. Data extraction and analysis was conducted in DistillerSR® (Evidence Partners Inc., Ottawa, Canada).

Descriptive tabulation of all pertinent information that aids in the characterisation and illustration of the available knowledge of the *Distribution, Frequency and Concentration of ESBL-producing Enterobacteriaceae of salmon and shrimp* were conducted in MS Excel (Microsoft Office, 2016). Meta-analysis or other statistical analyses were not conducted due to the scoping nature of this study and the heterogeneity in study data/methods.

## Results

**Fig. 1.**
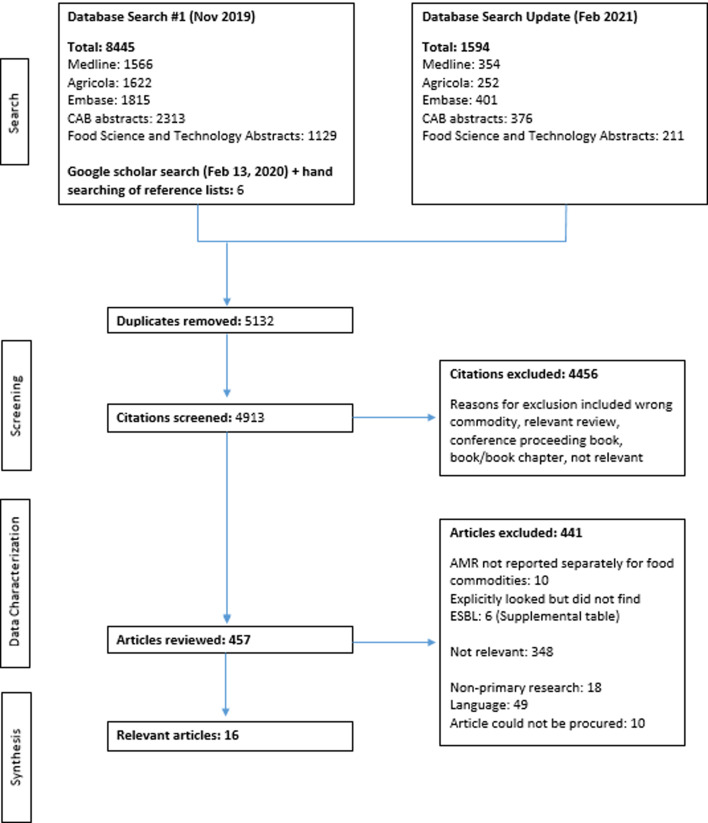
PRISMA flow diagram for scoping review of the distribution and frequency of extended-spectrum *β*-lactamase (ESBL)-producing Enterobacteriaceae in shrimp and salmon.

After a rigorous literature search and deduplication process, 4913 abstracts and titles were screened for relevance from which 457 full-text articles were reviewed. A total of 16 research articles on ESBL-E in salmon or shrimp were characterised in this ScR (Fig. 1. PRISMA Diagram). Exclusions were due to non-relevance to the research topic (*n* = 348), non-primary research (*n* = 18), language (*n* = 49) and inability to procure full-text article (*n* = 10). Six articles explicitly looked for ESBL-E (either phenotypically or genotypically) and none were identified, and therefore no outcomes were available for extraction. These were retained, however, for any future analysis or systematic review that would be completed on this topic (S2, Supplementary Material). Furthermore, 10 studies were excluded because they did not report AMR data separately for each food commodity (S3, Supplementary Material).

**Fig. 2.**
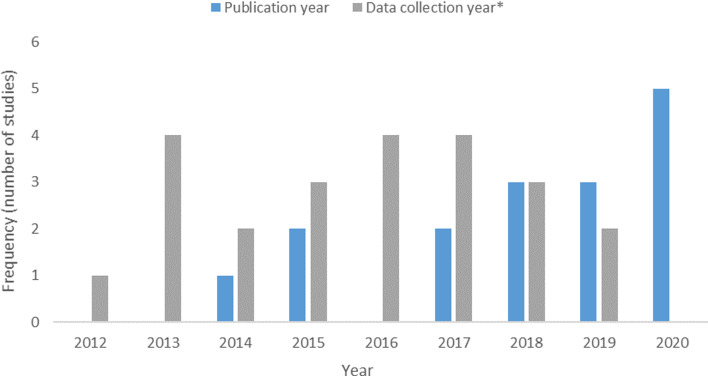
Histogram of the years of data collection and publication year for studies included in the scoping review of the distribution and frequency of extended-spectrum *β*-lactamase (ESBL)-producing Enterobacteriaceae in shrimp and salmon. Each year of data during a multi-year collection period is recorded as one value under frequency of data collection*.

Retained articles were published between 2014 and 2020 (Fig. 2. Histogram of Publication Dates). The time between isolate collection and publication ranged from 1 to 4 years. Studies were conducted in India (*n* = 3), Thailand (*n* = 2), Vietnam (*n* = 3), Myanmar (*n* = 1), Denmark (*n* = 1), Germany (*n* = 1), Algeria (*n* = 1), Portugal (*n* = 1), Spain (*n* = 1), Brazil (*n* = 1) and the United States (*n* = 1). Studies either reported the prevalence or frequency of ESBL-producing bacteria; none reported extractable concentrations. Studies were primary-research articles, cross-sectional in design and utilised a convenience sampling approach. No surveillance reports monitoring the occurrence of ESBL-E in these food commodities were identified in the ScR; however, it should be noted that grey literature was only investigated through a Google search in this review and not an intensive search of government/other grey literature sources.

The majority of included studies (*n* = 14) identified ESBL-E in shrimp/prawns while only 2 studies identified ESBL-E in salmon (Table 1). All of the samples were collected at the retail/market level and studies reported collection of fresh/raw samples (*n* = 7), frozen samples (raw or precooked, *n* = 2) and samples of unknown status (*n* = 5).

**Table 1.** Details of studies that reported the presence of extended-spectrum *β*-lactamase (ESBL)-producing Enterobacteriaceae in salmon and shrimp

Study	Source (species) Form(s) of sample	Country (Study Conducted)	Country origin of sample (Year samples collected)	Enterobacteriaceae species isolated (species positive for ESBL in bold)	Enterobacteriaceae frequency of ESBL positive isolate(s) n/N (%) (e.g., sample and/or isolate prevalence)	ESBL-E frequency n/N (%) (e.g., sample and/or isolate prevalence)	Resistance phenotype(s) identified	Resistance genotype(s) identified
[33]	Shrimp (*Penaeus* sp.) Retail	India	India (2019)	***K. pneumonieae E. coli***	*K. pneumoniae*: 1 isolate detailed *E. coli*: 2 isolates detailed	Phenotypic ESBL confirmation not conducted	AMP-ATM-CZ-FEP-CTX-CAZ-CIP-LVX(I)-PIP-SXT AMP-ATM-CZ-CTX-CIP(I)-LVX(I)-PIP	*K. pneumoniae*: *bla*_CTX−M−15_/*bla*_TEM_/*bla*_SHV_ (1 isolate) *E. coli*: *bla*_CTX−M−15_ (1 isolate)
[42]	Shrimp (*Metapenaeus dobsoni* and *Acetes indicus*) Retail, Fresh/raw	India	India (2016–2018)	***E. coli***	*M. dobsoni*: 26 isolates/3 samples tested *A. indicus*: 27 isolates/5 samples tested	*M. dobsoni*: 19/26 (73.7%) (ESBL-production detected in 19 of 26 isolates tested) *A. indicus*: 27/27 (100%) (ESBL-production detected in 27 of 27 isolates tested)	CIP-FOX-CTX-CAZ-ATM-IMP-AMC-CPD-MEM-PIT-ETP Note: Antibiotic resistance profile based on 2 isolates	*M. dobsoni*: *bla*_TEM_ (2/19, 10.5%) *bla*_SHV_ (2/19, 10.5%) *bla*_CTX−M_ (11/19, 57.9%) *A. indicus*: *bla*_CTX−M_ (23/27, 85.2%) *bla*_SHV_ (12/27, 44.4%)
[48]	Shrimp (*Macrobrachium lanchesteri*) Retail, Fresh/raw	Thailand	Thailand (2019)	*K. pneumoniae* ***P. penneri*** *P. vulgaris* *E. aerogenes*	23 isolates/80 samples tested	Phenotypic ESBL confirmation not conducted	AMP-CXM-PIT (I)	*bla*_CTX−M_ (isolates unclear)
[34]	Prawns (Penaeidae) Farmed, Retail, Fresh/raw, Ready to eat, Frozen	Denmark	Vietnam & Bangladesh (2017–2018)	***E. coli***	17 isolates/197 samples tested (8.6%)	Phenotypic ESBL confirmation not conducted	AMP-AZM-CHL-CIP-COL-FEP-CTX-FOX-GEN-NAL-SMX-CAZ-TET-TMP	*bla*_CTX−M−15_ (2/4 isolates) *bla*_CTX−M−55_ (2/4 isolates)
[47]	Prawn (unspecified) Retail	Myanmar	Myanmar (2017)	***K. pneumoniae***	NR	Phenotypic ESBL confirmation not conducted	GEN-CAZ IMP-AN-MEM-MOL	*bla*_CTX−M−15_ (1 isolate)
[35]	Shrimp (*Penaeus monodon*, *Litopenaeus vannamei*, *Penaeus merguiensis, Metapenaeus ensis, Macrobrachium rosenbergii*) Retail, Fresh/raw	Vietnam	Vietnam (2018)	**Non-typhoidal *Salmonella* (NTS):** Agona, Albany, Anatum, Bareilly, Bovismorbificans, **Braenderup**, Derby, Enteritidis, **Give**, **Infantis**, Kedougou, Kentucky, Litchfield, London, Ohio, Paratyphi B, Poona, Potsdam, Rissen, Saintpaul, Senftenberg, Stanley, Typhimurium, Urbana, Weltevreden	30/40 (75%) batches of shrimp (250–300 g each)	7/90 (7.8%) (ESBL-production detected in 7 isolates of 90 isolates tested) ESBL-producing organisms were identified as belonging to serovars Braenderup (1), Infantis (3), and Give (3)	CFM-CTX-CAZ-CRO-CIP-LVX(I)-MOX(I)-NAL-OFX-GEN-TOB-ATM-TIG-AMP-PIP-TIC-CHL-CEP-FOX(I)-CXM-CXM/A-MIN-TET-TMP-SXT-NIT	*S.* Infantis: *bla*_CTX−M−9_ (3 isolates from same sample) *S.* Give: *bla*_CTX−M−1_ and *bla*_TEM_ (3 isolates)
[46]	Shrimp (*P. monodon*) Farmed, Retail, Frozen	United States	Unspecified	***E. coli***	55 isolates/207 shrimp samples	Phenotypic ESBL confirmation not conducted	AMP-PEN-TET	*bla*_CTX−M_: 31/55 isolates *bla*_TEM_: 9/55 isolates *bla*_CTX−M_/*bla*_TEM_: 6/55 isolates
[41]	Shrimp (*P. monodon, L. vannamei*) Retail, Fresh/raw	Germany	Unknown, Vietnam, Bangladesh, India (2015–2016)	***K. pneumoniae*** *E. coli* *E. aerogenes* *E. cloacae* *M. morganii* *C. braakii* *C. freundii* *L. adecarboxylata*	NR	*P. monodon*: 15/55 (27.3%) (ESBL and AmpC production detected in 15 of 55 samples tested) *L. vannamei*: 3/25 (12%) (ESBL and AmpC production detected in 3 or 25 samples tested)	NR	*P. monodon* (E. coli): *bla*_CTX−M−15_ (2 isolates) *P. monodon* (K. pneumoniae): *bla*_CTX−M−9_/*bla*_SHV−1_/*bla*_DHA−1_ (1 isolate) *bla*_CTX−M−9_/*bla*_SHV−27_/*bla*_DHA−1_ (1 isolate) *bla*_CTX−M−9_/*bla*_SHV−62_/*bla*_DHA−1_ (1 isolate) *bla*_SHV−2a_/*bla*_TEM−1_ (1 isolate)
[36]	Shrimp (*Aristeus antennatus*) Retail	Algeria	Algeria (2015)	***E. coli***	66% of samples positive	1/4 (25%) (ESBL production detected in 1 of 4 isolates tested)	AMP-AMX-AMC-CEP-AN-KAN-GEN-N-TOB-TET	*bla*_CTX−M−15_ (1 isolate)
[43]	Shrimp (*A. indicus, M. dobsonii)* Retail, Fresh/Raw	India	India (2013–2014)	**Enterobacteria** (species isolated from shrimp not specified)	*A. indicus*: 12 isolates/1 sample tested *M. dobsonii*: 19 isolates/2 samples tested	*A. indicus*: 12/12 (100%) (ESBL production detected in 12 of 12 isolates tested) *M. dobsonii*: 14/19 (74%) (ESBL production detested in 14 of 19 isolates tested)	Resistance phenotypes were not reported separately for shrimp samples	All the isolates harboured two or more ESBL genes (*bla*_CTX_, *bla*_TEM_ or *bla*_SHV_) but this study did not report resistance genes for shrimp samples specifically (genes results were combined across all seafood species tested).
[39]	Shrimp (*L. vannamei*) Farmed, Retail, Fresh/Raw	Brazil	Brazil (NR)	*E. coli* *K. pneumoniae* *E. aerogenes* ***E. cloaceae*** *C. freundii*	5 samples positive/5 samples tested (100%) 25 isolates/5 samples tested	2/25 (8%) (ESBL production detected in 2 of 25 isolates)	CTX-CRO-CAZ-FEP-PEN	NR
[37]	Shrimp (unspecified) Retail	Vietnam	Vietnam (2013–2014)	***E. coli***	NR	45/60 (75%) (ESBL production detected in 45 of 60 samples tested) 85/60 (ESBL production detected in 85 isolates of 60 samples)	AMP-CHL-CIP- NAL-STR-TET-GEN-KAN-FOX-CTX-CAZ-SXT-FOF	*bla*_TEM_ (6/85 isolates) *bla*_CTX−M−1_ (9/85 isolates) *bla*_CTX−M−9_ (35/85 isolates) *bla*_CTX−M−1_/*bla*_TEM_ : 13/85 *bla*_CTX−M−9_/*bla*_TEM_: 22/85
[38]	Shrimp (unspecified) Retail	Vietnam	Vietnam (2013)	***E. coli***	NR	11/60 (18.3%) (ESBL production detected in 11 isolates of 60 samples)	AMP-CHL-CIP-NAL-TET-GEN-KAN-STR-CTX-CAZ-SXT-FOF	*bla*_CTX−M−1_: 3/11 isolates *bla*_CTX−M−1_/*bla*_TEM_: 3/11 isolates *bla*_CTX−M−1_/*bla*_SHV_/*bla*_TEM_: 1/11 isolates *bla*_CTX−M−9_: 1/11 isolates *bla*_CTX−M−9_/*bla*_SHV_: 1/11 isolates *bla*_CTX−M−9_ + *bla*_TEM_: 2/11 isolates
[40]	Shrimp (species unspecified) Retail, Fresh/raw	Thailand	Thailand (2012–2013)	***Klebsiella* spp.** *Enterobacter* spp.	5 samples positive/10 samples tested (50%)	1/5 (20%) (ESBL production detected in 1 sample of 5 samples tested)	NR	NR
[44]	Salmon (*Salmo salar*) Retail, Fresh/raw	Portugal	Portugal (2016–2017)	***E. coli***	15 isolates/30 samples tested	1/15 (6.7%) (ESBL-production detected in 1 of 15 isolates tested)	AMP-STR-CTX-CAZ	*bla*_CTX−M−1_/*bla*_TEM_ (1 isolate)
[45]	Salmon (unspecified) Retail, Fresh/raw	Spain	Spain (2015–2016)	**Enterobacteriaceae (unspecified)**	NR	1/28 (3.6%) (ESBL-production detected in 1 sample of 28 samples tested)	NR	None found

AMC, Amoxicillin-clavulanic acid; AMG, Aminoglycosides; AMP, Ampicillin; AMX, Amoxicillin; AN, Amikacin; APL, Amphenicol; ATM, Aztreonam; AZM, Azithromycin; BAC, Bacitracin; CAZ, Ceftazidime; CEC cefaclor; CEP, Cephalothin; CFM, Cefixime; CFP, Cefoperazone; CHL, Chloramphenicol; CIP, Ciprofloxacin; CLA, Clavulanic acid; CLI, Clindamycin; COL, Colistin; CPD, Cefpodoxime; CPM, carbapenem; CRO, Ceftriaxone; CTX, Cefotaxime; CXM, Cefuroxime; CXM/A, Cefuroxime Axetil; CZ, Cefazolin; DOX, Doxycycline; EN, Enrofloxacin; ERY, Erythromycin; ETP, Ertapenem; FEP, Cefepime; FFC, Florfenicol; FIS, Sulfisoxazole; FLQ, Fluoroquinolones; FOF, Fosfomycins; FOX, Cefoxitin; GEN, Gentamicin; IMP, Imipenem; KAN, Kanamycin; LVX, levofloxacin; LZD, linezolid; MAC, Macrolides; MEM, Meropenem; MEZ, Mezlocillin; MIN, Minocycline; MOL, Moxalactam; MOX, Moxifloxacin; N, Neomycin; NAL, Nalidixic acid; NIT, Nitrofurantoin; NOR, norfloxacin; NOV, Novobiocin; OFX, ofloxacin; OTC, Oxytetracycline; PEN, Penicillin; PIP, Piperacillin; PIT, Piperacillin/Tazobactam; PMB, polymyxin B; RIF, Rifampicin; SAM, Ampicillin/sulbactam; SMX, Sulfamethoxazole; SPC, Spectinomycin; SSS, Sulfisoxazole; STR, Streptomycin; SXT, Trimethoprim-sulfamethoxazole; TEL, Telithromycin; TET, Tetracycline; TIC, Ticarcillin; TIG, Tigecycline; TIO, Ceftiofur; TOB, Tobramycin; TMP, Trimethoprim; TZB, Tazobactam; VAN, Vancomycin; NR, Not reported; I, Intermediate Resistance.

### Phenotypic results

Among the included studies, 15/16 used selective media and/or antimicrobial susceptibility testing results to identify potential ESBL phenotypes. Double disk diffusion/double-disk synergism was conducted in 11 studies to confirm ESBL production [32]. When specified, CLSI (CLSI guideline (M100). Wayne, PA: Clinical and Laboratory Standards Institute) cut-points were used for susceptibility classification in 9 studies and EUCAST (European Committee on Antimicrobial Susceptibility Testing. Breakpoint tables for interpretation of minimum inhibitory concentrations and zone diameters. http://www.eucast.org/clinical_breakpoints/) were used in 2 studies. While not all studies may have been designed to look for resistance to multiple antimicrobial classes, 6 studies reported isolates resistant to >2 antimicrobial classes [33–38] and 4 reported resistance to >4 antimicrobial classes [34, 35, 37, 38].

Phenotypic detection of ESBL-E included: *Enterobacter cloaceae* in shrimp (*L. vannamei*) from Brazil [39], *Klebsiella* spp. in shrimp from Thailand [40], *K. pneumoniae* and *E. coli* in shrimp (*Penaeus monodon* and *L. vannamei*) from Vietnam, Bangladesh and India [41], *E. coli* in shrimp (*Aristeus antennatus*) from Algeria [36], *E. coli* in shrimp from Vietnam [37, 38], *E. coli* in shrimp (*Metapenaeus dobsoni* and *Acetes indicus*) from India [42], non-typhoidal *Salmonella* in shrimp from Vietnam [35], unspecified Enterobacteria in shrimp (*M. dobsoni* and *A. indicus)* from India [43], *E. coli* in salmon (*S. salar*) from Portugal [44] and unspecified Enterobacteriaceae in salmon from Spain [45].

### Genotypic results

Several studies reported on single- and multi-ESBL genes in isolates derived from shrimp and salmon samples, identified via polymerase chain reaction and whole genome sequencing. However, they did not always report the specific gene type for the identified *bla*_TEM_, *bla*_SHV_ and *bla*_CTX−M_ genes. Because not all *bla*_TEM_ and *bla*_SHV_ genes encode ESBLs, a decision was made to only include unspecified *bla*_TEM_/*bla*_SHV_ genes when another ESBL gene like *bla*_CTX−M_ was identified in the study or phenotypic prevalence of ESBL production was detected in the study.

Three studies detected ESBL-E genotypes in imported seafood products (Table 1). ESBL genes *bla*_CTX−M−15_ and *bla*_CTX−M−55_ were detected in *E. coli* isolated from prawns purchased in Denmark that were imported from Vietnam and Bangladesh [34]. Single- and multi-ESBL genes were detected in shrimp purchased in Germany that were imported from Vietnam, Bangladesh and India including *E. coli* with *bla*_CTX−M−15_ and *K. pneumoniae* isolates with *bla*_CTX−M−9_/*bla*_SHV−1_/*bla*_DHA−1_, *bla*_CTX−M−9_/*bla*_SHV−27_/*bla*_DHA−1_, *bla*_CTX−M−9_/*bla*_SHV−62_/*bla*_DHA−1_ and *bla*_SHV−2a_/*bla*_TEM−1_ [41]. Single- and multi- ESBL genes were also detected in *E. coli* isolated in imported shrimp (origin not specified) in the United States including *bla*_CTX−M_, *bla*_TEM_ and *bla*_CTX−M_/*bla*_TEM_ [46].

The majority of ESBL genes detected in shrimp were in Enterobacteriaceae isolated from samples collected in Vietnam (Table 1). Single- and multi-ESBL genes were detected in *E. coli* isolates and included *bla*_TEM_, *bla*_CTX−M−1_, *bla*_CTX−M−9_, *bla*_CTX−M−1_/*bla*_TEM_, *bla*_CTX−M−9_/*bla*_TEM_, *bla*_CTX−M−9_/*bla*_SHV_, *bla*_CTX−M−1_/*bla*_SHV_/*bla*_TEM_ [37, 38]. Also detected were *bla*_CTX−M−9_ and *bla*_CTX−M−1_/*bla*_TEM_ in *Salmonella* Infantis and *S.* Give isolated from shrimp in Vietnam, respectively [35].

In India, ESBL genes detected in shrimp included *bla*_TEM_, *bla*_SHV_ and *bla*_CTX−M_ from *E. coli* [42] and *bla*_CTX−M−15_ and *bla*_CTX−M−15_/*bla*_TEM_/*bla*_SHV_ from *E. coli* and *K. pneumoniae*, respectively [33] (Table 1).

Other genes identified included *bla*_CTX−M−15_ from *E. coli* isolated in shrimp from Algeria [36], *bla*_CTX−M−15_ from *K. pneumoniae* isolated in shrimp from Myanmar [47] and *bla*_CTX−M_ from *P. penneri* in shrimp from Thailand [48]. The only study that identified an ESBL gene from salmon detected *bla*_CTX−M−1_/*bla*_TEM_ in an *E. coli* isolate in a sample from Portugal [44]. The bacterial species and genes identified are summarised in Table 2.

**Table 2.** Synthesis table of ESBL genes found in Enterobacteriaceae from salmon and shrimp

Food species	Bacterial species identified with ESBL genes (# articles)	ESBL genes found	Studies
Salmon	*Escherichia coli* (*n* = 1)	*bla*_CTX−M−1_, *bla*_TEM_	[44]
Shrimp	*E. coli* (*n* = 8)	*bla*_CTX−M−15_, *bla*_TEM_, *bla*_SHV_, *bla*_CTX−M_, *bla*_CTX−M−55_, *bla*_CTX−M−1_, *bla*_CTX−M−9_	[33, 34, 36–38, 41, 42, 46]
	*Klebsiella pneumoniae* (*n* = 3)	*bla*_CTX−M−15_, *bla*_CTX−M−9_, *bla*_SHV−27_, *bla*_SHV−2a_, *bla*_TEM_	[33, 41, 47]
	*Proteus penneri* (*n* = 1)	*bla*_CTX−M_	[48]
	*Salmonella* Infantis (*n* = 1)	*bla*_CTX−M−9_	[35]
	*S*. Give (*n* = 1)	*bla*_CTX−M−1_, *bla*_TEM_	[35]

## Discussion

Our ScR found that *E. coli*, *K. pneumoniae,* and other *Enterobacteriaceae* producing ESBLs have been isolated in retail shrimp products originating in the Asian region. Only three studies reported ESBL-E in imported food products, two in Europe and one in the United States. This ScR found that there is a lack of published Canadian data on ESBL-E in salmon and shrimp. Research and surveillance activities regarding ESBL-E in Canadian retail seafood have been undertaken; the results of these studies are in the process of being analysed and disseminated (Personal communication with Janecko *et al*. [20]). Publication of this work will help address the data gap with AMR/ARGs in retail seafood in Canada. However, other important ARGs have been identified. One excluded Canadian study identified carbapenem-resistant Enterobacteriaceae in 8 of 1328 seafood samples investigated as part of a targeted retail surveillance project by the Public Health Agency of Canada [20]. Of 928 shrimp samples, 2 imported from Vietnam contained *E. cloacae* harbouring blaIMI − 1, and 1 from Bangladesh contained *E. aerogenes* harbouring blaIMI-2 [20]. While no ESBL-E was identified in shrimp, 2 clam samples from Vietnam contained *E. cloacae* harbouring *bla*_NDM−1_, *bla*_TEM_ and *bla*_OXA−1_ and were phenotypically resistant to ampicillin, cefoxitin and amoxicillin/clavulanic acid [20]. In another study, Janecko *et al*. found that imported shrimp were the source of over half (65%, 37/57) of all ESBL-E isolates identified in imported and domestic seafood products purchased at retail in Canada (*Personal communication* with Janecko *et al*. [20]). ESBLs were harboured by *E. coli*, *K. pneumoniae* and *Enterobacter* sp., with *bla*_CTX−M_ genes (*bla*_CTX−M−15_, *bla*_CTX−M−27_ and *bla*_CTX−M−55_) being the most common ESBL determinant in shrimp and seafood (*Personal communication* with Janecko *et al*.). Together, these findings indicate that contaminated retail seafood products in Canada may be a source of human exposure to AMR-organisms.

Across the published studies, there was a general and consistent lack of reporting of study methodology and results of interest to our ScR, precluding the ability to conduct a systematic review and/or meta-analysis on this specific study question. Some heterogeneity may be attributable to different study locations, sampling methods and test methods used for measuring bacterial prevalence and AMR. Some studies reported overall sample prevalence while others reported isolate prevalence; making direct comparisons and synthesis difficult. The reporting of the raw quantitative data concerning sample types and isolates as well as the genetic sequences obtained from PCR or whole genome sequencing analysis should be encouraged. Provided as supplemental information, it would enable inter-study comparison, even if the results as published are not evaluated using the same methodology. Additionally, the use of standard laboratory procedures for bacterial isolation where possible, and international standards for antimicrobial susceptibility testing or epidemiological cut-off values to evaluate antimicrobial susceptibility would permit the comparison of study results derived from different sample types or regions. Further limitations of the studies evaluated here included small sample sizes and convenience sampling instead of random sampling, which would have provided better representativeness of the information across the shrimp and salmon populations. We considered whether the taxonomy change which resulted in a new scientific order, ‘Enterobacterales’ in 2020 could be a limitation affecting our results; the only impact being that some species we had considered under the umbrella of Enterobacteriaceae moved to their own families (i.e., *Morganella* and *Providencia* are now under the Morganellaceae family and *Yersinia* and *Serratia* are now under the Yersiniaceae family) [26]. These all would have been captured by our study.

Our ScR has some limitations related to resourcing information on an emerging topic. Figure 2 demonstrates an increasing tendency in the number articles evaluating ESBL in seafood from 2012–2020. If the excluded publications presented in S2 and S3 in the Supplementary Materials had been included in this graphic, this tendency would be accentuated. This may speak to the increased interest in AMR research by Universities, Governments and International organisations [49, 50]. The top six countries exporting seafood to Canada are the US, China, Vietnam, Chile, Thailand and India respectively [51]. Among the 16 studies cited in this paper, India, Vietnam, Thailand and the US are present with 3, 3, 2 and 1 publications respectively. Additionally the US study examines shrimp imported into the United States, so the results would not be indicative of the local AMR environment/situation. The top two exporters of seafood to Canada, the US and China, were absent. Further, the US and Chile are the top exporters of salmon to Canada, however, no studies examining AMR in salmon were identified in this ScR [51]. Even if consideration were given to the 6 articles excluded for negative results and the 10 for which the reporting did not sufficiently stratify the seafood types, it would encompass 3, 4 and 1 additional articles attributed to the source countries of China, Vietnam and Chile respectively. The lack of proportional representativity of studies concerning countries exporting seafood to Canada belies an important data gap. The ScR and risk profile approach are time and resource demanding. We acknowledge that further relevant research on this topic has been published since the search strategy for this review was executed. After conducting this review we identified 3 studies that are worth mentioning here for further context, discussion and to further supplement the findings. Because these studies were not identified in a systematic manner we are unable to say that they represent the totality of relevant research published on this topic since our search was executed. One study from India reported that out of 77 shrimp samples, 2 tested positive for ESBL-*E. coli* and 4 for ESBL-*K. pneumonia* [52]. The genes *bla*_CTX−M−1_ and *bla*_CTX−M−9_ were each identified in 6 *E. coli* isolates from shrimp; *bla*_TEM_ and *bla*_SHV_ genes were isolated from 10 *K. penumoniae* isolates [52]. A study from Vietnam identified ESBL-*E. coli* in 19.6% (22/112) of retail shrimp examined [53]. Finally, a study from the United States found *bla*_CTX−M_ in 42% and *bla*_SHV_ genes in 6% of domestic wild caught imported shrimp; no ESBL genes were found in farmed shrimp imported from Ecuador [54]. However, pragmatism and timeliness are needed for making synthesis studies of ESBL-E available for decision-making; hence, this ScR provides useful and relevant information needed for current decision-making.

While the studies may not have been originally designed to detect resistance to multiple classes of antimicrobials, this ScR identified 6 studies reported isolates resistant to >2 antimicrobial classes [33–38] and 4 reported resistance to >4 antimicrobial classes [34, 35, 37, 38]. The use of *β*-lactam antimicrobials or other non-*β*-lactam antimicrobials such as aminoglycosides or (fluoro)quinolones administered via water or in-feed medications has the potential to select or enrich multidrug-resistant bacterial species carrying relevant co-resistance genetic determinants [29, 55] ESBL producers often display multidrug resistance against several important antimicrobial groups due to the co-presence of ESBL genes and other AMR genes in the same plasmid [41, 56–58]. Cross-resistance and co-resistance can facilitate the selection and enrichment of multidrug resistant bacteria. Many other factors are also known to motivate the emergence of AMR in aquaculture including high stocking densities, elevated stress and infections, chemical additives, nutrient-rich environments and release of human waste into the environment [28].

In terms of current regulations in the aquaculture industry, cephalosporins are not authorised for use in aquaculture operations in the European Union or North America [59–61]. However, their use has been reported in Asia including 1st generation cephalosporins (cephalexin and cefradine) in Vietnam and China and cefotaxime, a 3rd generation cephalosporin, detected in Chinese aquaculture operations [25, 62–64]. In Canada, there are a small number of non-*β*-lactam antimicrobials authorised for use in aquaculture including erythromycin, florfenicol, ormetoprim, oxytetracycline and sulphonamides [65]. Regulatory structure and enforcement may differ among aquaculture producing countries, as well as the antimicrobial classes used. There remains a lack of regulatory structure and enforcement in several countries known to export seafood to Canada [29]. Canada has established risk management regulations such as Safe Food for Canadians Regulations which includes a preventative control plan for seafood importations [66]. Although this system can help maintain low levels of antimicrobial residues in relation to imported aquacultured products, it does not include an evaluation of AMR. Moving forward, surveillance using modern molecular techniques such as PCR and whole genome sequencing would be useful in detecting AMR genes of concern in imported seafood products. These are just brief considerations; a more comprehensive evaluation of aquaculture operations and regulations will be conducted as part of the risk profile.

Together, the findings presented in this review indicate that seafood products from countries with a history of exporting to Canada have been identified to harbour ESBL-producing Enterobacteriaceae. Salmon and shrimp would be appropriate initial targets for surveillance of seafood AMR in Canada, as they are the two most commonly consumed seafood products in Canada and would provide a snapshot of both domestic and imported seafood product for the distribution, frequency and concentrations of this AMR hazard(s) in the food chain. Genetic analysis of bacteria is of utmost importance in the risk analysis process. Understanding the interconnection between bacterial species, AMR genotypes and mobile genetic elements (i.e., plasmids) will be vital to the understanding of AMR selection and dissemination, particularly under a ‘One Health’ lens. Our intention is that the available literature described herein will support decision-making on this issue as a component of a larger risk profile.

## Data Availability

Materials needed to replicate the findings of the article are available as Supplementary Materials.

## Supplementary material

For supplementary material accompanying this paper visit http://doi.org/10.1017/S0950268822001819. click here to view supplementary material
