# Supplementary material for: A scoping review of the distribution and frequency of extended-spectrum β-lactamase (ESBL)-producing Enterobacteriaceae in shrimp and salmon
Source: Epidemiol Infect. 2022 Dec 5;151:e1. doi: 10.1017/S0950268822001819 (PMC9990388; doi:10.1017/S0950268822001819)
Supplement: Supplementary file 1 [file S0950268822001819sup001.docx]

*Epidemiology and Infection*

A Scoping Review of the Distribution and Frequency of Extended Spectrum β-lactamase (ESBL)-Producing Enterobacteriaceae in Shrimp and Salmon

K.M. Young ^1^, M.J. Isada ^2^, M. Reist ^3^, F. C. Uhland ^2^, L. Sherk ^2^, C. Carson ^2^.

# **Supplementary Material**

## S1: Protocol for a Scoping Review of the Distribution and Frequency of Extended Spectrum β-lactamase (ESBL)-Producing Enterobacteriaceae in Shrimp and Salmon

## Scope: Extended-spectrum β-lactamase-producing Enterobacteriaceae of salmon and shrimp origin

## Authors and Affiliations:

K.M. Young ^1^, M. Isada ^2^, M. Reist ^3^, F. C. Uhland ^2^, L. Sherk ^2^, C. Carson ^2^.

^1^ Public Health Risk Sciences Division, National Microbiology Laboratory, Public Health Agency of Canada, Guelph, Ontario, Canada.

^2^ Centre for Food-borne, Environmental and Zoonotic Infectious Diseases, Public Health Agency of Canada, Guelph, Ontario, Canada.

^3^ Veterinary Drugs Directorate, Health Products and Food Branch, Health Canada, Ottawa, Ontario, Canada

Contact: Kaitlin Young, Email: [kaitlin.young@phac-aspc.gc.ca](mailto:kaitlin.young@phac-aspc.gc.ca)

## Important Dates:

Evidence published up to October 31, 2019.

Protocol version 1, initiated October 22, 2019.

Table of Contents

[Extended Spectrum β-lactamase (ESBL) producing bacteria in seafood risk profile using the Codex Framework 1](#_Toc63943289)

[Scope: Extended-spectrum β-lactamase-producing Enterobacteriaceae of salmon and shrimp origin 1](#_Toc63943290)

[Authors and Affiliations: 1](#_Toc63943291)

[Important Dates: 1](#_Toc63943292)

[Rationale 2](#_Toc63943293)

[Background 3](#_Toc63943294)

[References 3](#_Toc63943295)

[Study Question 4](#_Toc63943296)

[Planned Study Outputs 4](#_Toc63943297)

[**Methods** 4](#_Toc63943298)

[Review Team Expertise and Responsibilities 4](#_Toc63943299)

[Search Strategy 4](#_Toc63943300)

[Algorithms 4](#_Toc63943301)

[Databases 4](#_Toc63943302)

[Grey Literature Sources and Procedures 5](#_Toc63943303)

[Search Verification 5](#_Toc63943304)

[Relevance Screening 6](#_Toc63943305)

[Inclusion/Exclusion criteria 6](#_Toc63943306)

[Study Characterization 6](#_Toc63943307)

[Review Management 6](#_Toc63943308)

[Data Analysis 6](#_Toc63943309)

[Appendix 1: Relevance Screening Tool 6](#_Toc63943310)

[Appendix 2: Data Characterization and Utility (DCU) Form 7](#_Toc63943311)

[Appendix 3: Search Strategy Implemented 14](#_Toc63943312)

[Appendix 4: Search Strategy Updated in 2021 18](#_Toc63943313)

## Rationale

A foodborne AMR risk profile presents prerequisite scientific information on identified food safety issues to inform risk managers prior to decision-making. A risk profile incorporates various elements for consideration including information on antimicrobial resistant microorganism(s) and/or determinant(s). A scoping review will be conducted to review current literature for section 2.1.4 of the Codex risk profile guidelines: Distribution, frequency and concentrations of the AMR hazard(s) in the food chain. This project has been prioritized by via expert/stakeholder consultation as an important AMR hazard topic relevant to food safety in Canada.

## Background

Antibiotic-resistant bacteria is a growing threat to public health as they have the ability to find new ways to resist treatment and can pass along genetic material that allows other bacteria to become drug-resistant (WHO, 2017). In 2017, the World Health Organization published its first ever list of antibiotic-resistant “priority pathogens” that pose the greatest threat to human health (WHO, 2017). Priority 1 was considered critical and included carbapenem-resistant and extended spectrum β-lactamase (ESBL)-producing Enterobacteriaceae (WHO, 2017). These bacteria can cause severe infections and have become resistant to many antibiotics, including the best available treatments for multi-drug resistant bacteria (WHO, 2017).

Enterobacteriaceae that possess and express carbapenemase and ESBL enzyme-encoding genes have been identified in isolates of retail seafood in Canada (Janecko et al., 2016; Mangat et al., 2016). The Canadian Integrated Program for Antimicrobial Resistance Surveillance (CIPARS) monitors major meat commodities for antimicrobial resistance (AMR), where targeted studies with methods based on core surveillance protocols are used to examine other foods such as seafood (Mangat et al., 2016). The Government of Canada is conducting risk profiles of select antimicrobial-resistant bacteria originating from food of animal-origin according to the international Codex Alimentarius Guidelines for Risk Analysis of Foodborne AMR (CAC/GL 77- 2011). The objective of a risk profile is to put the situation into context and to make a recommendation to policy makers including evaluation of the potential public health risks. These findings have implications to decisions concerning the need for ongoing surveillance of AMR in seafood and options for risk management. Several elements within a risk profile lend themselves to scoping reviews. One of the risk profile elements that often may require a detailed scoping review is the distribution, frequency and concentration of the AMR hazard in the food chain.

### References

**Food and Agriculture Organization of the United Nations** (2011). Codex guidelines for the risk
 analysis of foodborne antimicrobial resistance (CAC/GL 77-2011). Retrieved from
 http://www.codexalimentarius.net/download/standards/11776/CXG_077e.pdf

**Janecko N, et al.** (2016). Carbapenem-Resistant Enterobacter spp. in Retail Seafood Imported from
 Southeast Asia to Canada. *Emerging infectious diseases*,***22***, 1675–1677.
 doi:10.3201/eid2209.160305

**Mangat CS, et al.** (2016). Characterization of VCC-1, a Novel Ambler Class A Carbapenemase from
 Vibrio cholerae Isolated from Imported Retail Shrimp Sold in Canada. *Antimicrobial
 agents and chemotherapy*, ***60***, 1819–1825. doi:10.1128/AAC.02812-15

**World Health Organization** (WHO). (2017). WHO publishes list of bacteria for which new
 antibiotics are urgently needed. Retrieved from https://www.who.int/news-
 room/detail/27-02-2017-who-publishes-list-of-bacteria-for-which-new-antibiotics-are-
 urgently-needed.

## Study Question

- **Codex question 2: Information on AMR microorganism(s) and/or determinant(s)**

1. Characteristics of the identified foodborne microorganism(s)
   - 1. Distribution, frequency and concentrations of the AMR hazard(s) in the food chain

## Planned Study Outputs

- Complete a scoping review, following standard methods, of select aspects of the Codex Alimentarius Guidelines for Risk Analysis of Foodborne Antimicrobial Resistance (CAC/GL 77- 2011).

## **Methods**

## Review Team Expertise and Responsibilities

| Member | Organization | Role |
| --- | --- | --- |
| Carolee Carson | CIPARS/CFEZID | Oversight |
| Kaitlin Young | CIPARS/CFEZID | Synthesis expertise/oversight/reviewer |
| Mark Reist | CIPARS/CFEZID | Synthesis expertise/oversight/reviewer |
| Melissa Isada | CIPARS/CFEZID | Reviewer |
| Lauren Sherk | CIPARS/CFEZID | Reviewer |
| Carl Uhland | CIPARS/CFEZID | Reviwer |

## Search Strategy

## Algorithms

See Appendix 3 and 4.

## Databases

- - Medline
  - Embase
  - Agricola
  - CAB Abstracts
  - Food Science and Technology Abstracts

## Search Verification and Grey Literature Sources and Procedures

**Total Articles Pre-deduplication:**

- Medline: 1566
- Agricola: 1622
- Embase: 1815
- CAB abstracts: 2313
- Food Science and Technology Abstracts: 1129

**Total Pre-deduplication: 8,445**

**Total after 1^st^ round de-duplication (in EndNote): 4,403**

**Search Verification:** The electronic search will be verified by hand searching the reference lists of three literature reviews focused or partially focused on AMR in salmon or shrimp:

1. **Thornber, K. , Verner‐Jeffreys, D. , Hinchliffe, S. , Rahman, M. M., Bass, D. and Tyler, C. R. (2019). Evaluating antimicrobial resistance in the global shrimp industry. Rev Aquacult. doi:10.1111/raq.12367**

-Akinbowale OL, Peng H, Barton MD (2006) Antimicrobial resistance in bacteria isolated from aquaculture sources in Australia. Journal of Applied Microbiology 100: 1103– 1113.

1. **Cabello, F., Godfrey, H., Tomova, A., Ivanova, L., Dölz, H., Millanao, A., & Buschmann, A. (2013). Antimicrobial use in aquaculture re‐examined: Its relevance to antimicrobial resistance and to animal and human health. Environmental Microbiology, 15(7), 1917-1942.**

-Miranda, C., & Zemelman, R. (2002). Antimicrobial multiresistance in bacteria isolated from freshwater Chilean salmon farms. Science of the Total Environment, 293(1-3), 207-218.

1. **Miller RA, Harbottle H. Antimicrobial Drug Resistance in Fish Pathogens. Microbiology spectrum. 2018;6(1).**

-None

**Google Scholar Search (Feburary 13th, 2020)**

The following search string was ran in Google Scholar and retrieved 100 results:

(antibiotic resistance) and (ESBL) and (Enterobacteriaceae) and (salmon or shrimp)

To limit the amount of irrelevant articles in our search, only articles that mentioned antimicrobial resistance were considered for inclusion into level 1 primary screening. In total, 24 articles were imported into Distiller for primary screening from the Google Scholar search. A total of 19 articles were excluded from primary screening and 5 articles were included for level 2 screening.

## Relevance Screening

### Inclusion/Exclusion criteria

1. Time frame- no time frame
2. Country – All
3. Language – English and French
4. Document Type – journal articles, PhD/MSc Theses, reports, conference papers
5. Agent/Disease: ESBL producing Enterobacteriaceae
6. Study design – All
7. Primary research: all articles describing the distribution, frequency, or concentration of ESBL producing enterobactericeae in salmon or shrimp will be included. Literature reviews will be identified for search verification.

Relevance Screening

Relevance screening tool included in appendix.

## Study Characterization

Study characterization tool included in appendix. The study characterization form will aim to classify and characterize the research on ESBL producing *Enterobactericeae* from salmon and shrimp. The form will first confirm the relevance of the publication prior to extracting important characteristics of the study. This will include language, location, study date, document type, focus of paper (i.e., relevant to ESBLs in the food chain), food commodity, if AMR data is extractable for each food commodity (exclude if not possible), and what Enterobactericeae were investigated, outcomes.

## Review Management

The search strategy will be exported and de-duplicated in Mendeley. These hits will then be exported to Rayyan, a web-based systematic review software. Relevance screening (keyword and abstract only) will be conducted in Rayyan. Full text of articles deemed relevant will then be procured. Data extraction and analysis will then be conducted in DistillerSR.

## Data Analysis

Descriptive tabulation of all pertinent information that aids in the characterisation and illustration of the available knowledge the Distribution, Frequency, and Concentration of ESBLs in *Enterobactericeae* of salmon and shrimp will be conducted mainly in MS excel. Meta-analysis or other statistical analyses will not be conducted due to the scoping nature of this study and the heterogeneity it study data/methods we expect to collect.

## Appendix 1: Relevance Screening Tool

| Question | Options | Definitions |
| --- | --- | --- |
| Does this citation describe primary research on:  The frequency/presence of ESBLs^1^ in Enterobacteriaceae^2^ of salmon and shrimp^3^. |  Yes – relevant primary research (add label for shrimp, salmon, trout, and environment samples) | **Extended-spectrum beta-lactamase (ESBL):** Members of the family commonly express plasmid-encoded β-lactamases (e.g., TEM-1, TEM-2, and SHV-1), which confer resistance to penicillins but not to expanded-spectrum cephalosporins. ESBLs are beta-lactamases that hydrolyze extended-spectrum cephalosporins with an oxyimino side chain. These cephalosporins include cefotaxime, ceftriaxone, and ceftazidime, as well as the oxyimino-monobactam aztreonam. Other types include CTX-M beta-lactamases and OXA beta-lactamases.  ^1^Because some abstracts do not report null results/all antibiotics tested reviewers are to err on the side of caution and include any studies that discuss AMR in bacteria and commodity(ies) of interest.  **Enterobacteriaceae** are Gram-negative bacteria of a large family that includes *Escherichia coli*, *Salmonella*, *Klebsiella*, *Proteus*, *Enterobacter*, *Morganella*, *Shigella*, *Citrobacter*, *Serratia*, *Yersinia*, and *Providencia*.  ^2^Reviews should err on the side of caution and screen include any studies that are vague in their reporting of what bacteria are studied.  The term **shrimp** is used to refer to decapod crustaceans of the groups *Caridea* and *Dendrobranchiata* (includes prawns).  For our purposes, the term **Salmon** refers to the *Salmonidae* family of ray-finned fish. It includes salmon as well as trout, chars, freshwater whitefishes, and graylings, (shall receive their own label) which collectively are known as the salmonids. |
|  | No – relevant review (exclude) |  |
|  | No – other relevant non-primary literature (exclude) |  |
|  | No – wrong commodity (exclude) (Add label for environment samples) |  |
|  | No – conference proceedings book (exclude) |  |
|  | No – book or book chapter (exclude) |  |
|  | No – not relevant (exclude) (Add label for environment samples) |  |
|  |  |  |

## Appendix 2: Data Characterization and Utility (DCU) Form

| **Question** | **Options** | Annotations |
| --- | --- | --- |
| Relevance Verification | | |
| 1. What language is the article published in? [Radio] |  English   French   Other, specify (exclude) | Languages other than English/French will be excluded, but will be captured in the “Other, specify” category for relative counts. |
| 1. What type of document is this article? [Radio] |  Primary research in peer-reviewed journal   Grey literature with primary data (Government or research reports)   Thesis/Dissertations   Other non-primary literature (exclude) |  |
| 1. Verify the focus of this paper [Checkbox] |  Distribution, frequency, and concentrations of the AMR hazard(s) in the food chain   Not Relevant (exclude) | Does this study report on ESBLs in the food chain? **Extended-spectrum beta-lactamase (ESBL):** ESBLs are beta-lactamases that hydrolyze extended-spectrum cephalosporins with an oxyimino side chain. These cephalosporins include cefotaxime, ceftriaxone, and ceftazidime, as well as the oxyimino-monobactam aztreonam. ESBL genes include BlaTEM, BlaSHV, and BlaCTX-M. Although not all TEMs and SHVs are ESBLs.  If they don’t specify the type of TEM or SHV (particularily if phenotypic evidence of ESBL production) we include anyway and note that further analysis would need to be conducted since sequencing is essential to discriminate between non-esbl parent enzymes (TEM1, TEM2, and SHV2) and different variants of TEM or SHV ESBLs (TEM3 and SHV2). Note that this was a limitation across studies. |
| 1. Which food commodities were included in the study? [Checkbox] |  Salmon   Shrimp   Other, specify (exclude)   Unspecified Seafood (exclude)   Not Reported (exclude) | For the Other, specify category, list commodity and species in brackets, capitalize the first letter of the commodity. If multiple, list alphabetically.  If it is the only commodity (no salmon or shrimp) exclude  Some papers do not specify the commodity and say “seafood,” “fish,” or “shellfish.” In this case check Unspecified Seafood and exclude  Not reported, exclude  The term **shrimp** is used to refer to decapod crustaceans of the groups *Caridea* and *Dendrobranchiata* (includes prawns).  For our purposes, the term **Salmon** refers to the *Salmonidae* family of ray-finned fish. It includes salmon (include) as well as trout, chars, freshwater whitefishes, and graylings (exclude). |
| 1. Does the study report the presence of Extended Spectrum Beta-lactamases in the commodity of interest? |  Yes (shrimp or salmon)   Yes (ESBL in other commodity-exclude if relevant commodities not also selected)   No (None found) | In studies that explicility looked for ESBLs in *Enterobactericeae* from salmon or shrimp, the DCU form will be completed to at least Q 17. If they looked for ESBLs in *Enterobactericeae* from salmon or shrimp and were unsuccessful, the DCU form will be completed to Q17. |
| 1. Is AMR data reported seperately for each commodity? [Radio] |  Yes   No (exclude) | If they looked for ESBLs and none were found check “yes” here. |
| 1. Were Enterobacteriaceae investigated in the study? [Radio] |  Yes   No (exclude) | **Enterobacteriaceae** are Gram-negative bacteria of a large family that includes *Escherichia coli*, *Salmonella*, *Klebsiella*, *Proteus*, *Enterobacter*, *Morganella*, *Shigella*, *Citrobacter*, *Serratia*, *Yersinia*, and *Providencia*. |
| 1. Were any exclusion criteria selected from the above relevance verification questions?  \|  \|  \| \| --- \| --- \| |  Yes (exclude)   No | If yes, stop here and submit the form. Do not fill out any subforms.  If no, continue to General Information |
| General Information | | |
| 1. From what continent was the study conducted? [Checkbox] |  North America   South America   Europe   Asia   Africa   Antarctica   Australia |  |
| 1. From what country was the study conducted? [Checkbox] |  Canada   United States  ** Option to permanently add answer to question* | If the country is not on the list, permanently add the country. |
| 1. From what country did the sample originate (i.e. imports)? [Checkbox] |  Canada   United States   Not specified  ** Option to permanently add answer to question* | If the country is not on the list, permanently add the country. |
| 1. When was the article published? [Text] | Enter Year | Format: yyyy |
| 1. What year were the samples collected? [Text] | Enter Year | Format: yyyy- yyyy  -Include range if applicable  NR if not reported |
| 1. What are the author reported study objectives? [Text] | Enter Objectives | Objectives often located at the end of the introduction. Copy and paste verbatim |
| 1. What Enterobacteriaceae were investigatedin the study? (select all that apply, specify serovars alphabetically in textbox). [Checkbox] |  *Salmonella*   *Escherichia*   *Klebsiella*   *Shigella*   *Proteus*   *Enterobacter*   *Morganella*   *Citrobacter*   *Serratia*   *Yersinia*   *Providencia*   *Raoutella*   Coliforms   Other, specify  **Each option has text-box for serovar*  **Option to permanently add answer to question* | Write serovar in lower case, if multiple list alphabetically.  When permanently adding an answer follow the format above.  Bacteria where ESBLs were found and for which ESBLS were investigated but not found should be checked off here. Only bacteria with ESBLs should carry on into the tables. |
| Study Details – Distribution/Frequency/Concentration of *Enterobactericeae* & General AMR   \| 1. Describe the sample population where bacteria was reported, including AMR details *Add a row for each food commodity and form of sample \| \| --- \| | | |
| 1. What food commodity was sampled? [Radio] | - Salmon - Shrimp |  |
| 1. What species was sampled? [Radio] | - Giant tiger prawn (*Penaeus monodon*) - Whiteleg Shrimp (*Liptopenaeus vannamei*) - Atlantic salmon (*Salmo salar*) - Chinook salmon (*Oncorhynchus tshawytscha*) - Not specified   ** Option to permanently add answer to question* | If not listed, permanently add an answer, follow the format above. |
| 1. From what environment did the samples originate? [Radio] | - Freshwater - Marine - Not specified - Other, specify |  |
| 1. Is the sample farmed or wild caught? [Radio] | - Farmed - Wild Caught - Not Specified |  |
| 1. At what point in the food chain were the samples collected, including the # of sampling units [Radio] | - Farm level aquaculture (Includes wild seafood) - Transport - Processing - Retail/market products   ** Text box for each to specify # sampling units* | Include the # of sampling units. Example: samples were collected from 3 different retail markets  If not reported specify NR in the text box |
| 1. What was the form of the sample? [Checkbox] | - Fresh/Raw - Ready to eat - Frozen - Not specified - Other, specify |  |
| 1. What *Enterobactericeae*  were isolated from the sample? [Checkbox] |  *Salmonella*   *Escherichia*   *Klebsiella*   *Shigella*   *Proteus*   *Enterobacter*   *Morganella*   *Citrobacter*   *Serratia*   *Yersinia*   *Providencia*   *Raoutella*   Coliforms   Other, specify  **Each option has a drop down text-box for serovar*  **Option to permanently add answer to question*  **Enter sample or isolate prevalence/frequency for each bacteria* | Write serovar in lower case, if multiple list alphabetically.  When permanently adding an answer follow the format above.  **Nested Question:**  What was the sample prevalence/frequency of bacteria (Sample and/or Isolate, PLEASE SPECIFY)? (x/N (%), CI, concentration CFU) If reported, state units used, Enter NR if not reported  NOTE: Only check off bacteria for which ESBLs were found. |
| Study Details-AMR | | |
| 1. Please indicate which antimicrobials were investigated in this sample [Checkbox] | - Ampicillin - Chloramphenicol - Ciprofloxacin - Lomeofloxacin - Nalidixic acid - Norfloxacin - Oflaxcin - Nitrofuratoin - Streptomycin   **Option to permanently add answer to question* | If not listed, permanently add an answer, follow the format above. |
| 1. What were the methods used for ESBL detection ? [Checkbox] | - Disc Diffusion (phenotypic) - NARMS plate - PCR (genomic) - PFGE - WGS - Not specified   **Enter* MIC interpretive criteria *for answer* | **Nested Question:** What MIC interpretive criteria was used? [Radio]   - EUCAST - CLSI - Not specified |
| 19. Study Details – AMR Specific  *Add a row for each bacteria | | |
| 1. Specify which sample the AMR specific data is for (note: specify species) [Text] | Enter sample | Specify commodity and species (in brackets) |
| 1. What *Enterobactericeae* was investigated? [Radio] |  *Salmonella*   *Escherichia*   *Klebsiella*   *Shigella*   *Proteus*   *Enterobacter*   *Morganella*   *Citrobacter*   *Serratia*   *Yersini*a   *Providencia*   *Raoutella*   Other, specify  **Each option has a drop down text-box for serovar*  **Enter prevalence/frequency of resistance for the bacteria*  **Option to permanently add answer to question* | Write serovar in lower case, if multiple list alphabetically.  Nested Question: What is the prevalence/frequency of resistance reported for the bacteria? (# isolates resistant/# isolates tested)  NR if not reported  For “Other, specify” write the bacteria in the text box, and then serovar in the corresponding dropdown text.  If not listed, permanently add an answer, follow the format above. |
| 1. Please indicate which antimicrobials/antibiotics the ESBL producing isolates were resistant to [Checkbox] | - Ampicillin - Chloramphenicol - Ciprofloxacin - Lomeofloxacin - Nalidixic acid - Norfloxacin - Oflaxcin - Nitrofuratoin - Streptomycin - Tetracycline   **Option to permanently add answer to question* | If not listed, permanently add an answer, follow the format above. |
| 1. Was presumptive phenotypic ESBL testing conducted? [Radio] | - Yes, specify - No - None detected | Specify in text box |
| 1. What resistant genes were detected? (specify variants of ESBLs if reported) [Checkbox] | - TEM-derived - SHV-derived - CTX-M - OXA - GES - PER - BES-1 - CME-1 - VE-B-1 - SFO - Not Reported - None detected   **Option to permanently add answer to question* | Specify variants of ESBLs if reported in text box  If not listed, permanently add an answer, follow the format above.  Nested Question:  If reported, specify prevalence/frequency of the resistance gene/variant |
| 1. Specify location of the resistance genes [Radio] | - Plasmid - Chromosomal - Not Reported |  |

## Appendix 3: Search Strategy Implemented

This will include all the search string details and number of hits

**MEDLINE: SEARCHED NOVEMBER 1, 2019**

Database Field Guide Ovid MEDLINE(R) and Epub Ahead of Print, In-Process & Other Non-Indexed Citations, Daily and Versions(R) 1946 to October 31, 2019

| **#** | **Searches** | **Results** |
| --- | --- | --- |
| 1 | exp *Drug Resistance, Microbial/ or exp *Drug Resistance, Multiple/ | 73608 |
| 2 | (resistan* or antimicrobial* or microbial* or antibiotic* or anti biotic* or antibacterial* or multidrug* or multi drug* or extensively drug or multiple drug* or multiclass* or multi class* or multiple class* or extended-spectrum or CTX-M or TEM or SHV).ti,ab,kw,kf | 1491930 |
| 3 | exp *beta-Lactams/ | 80165 |
| 4 | (ESBL or "B-lactam*" or "beta-lactam*" or penicillin* or carbapenem* or cephalosporin* or moxalactam* or latamoxef*).ti,ab,kw,kf. | 112344 |
| 5 | or/1-4 [AMR] | 1556547 |
| 6 | (fish or aquaculture or aquafarm* or fishfarm* or aquarium* or seafood or shellfish or crustacean* or shrimp* or prawn* or salmon or salmonid or salmonids).ti,ab,kw,kf. | 200714 |
| 7 | Escherichia coli.sh,xs. or Escherichia coli.ab,kf,ti. or e coli.ab,kf,ti. | 384402 |
| 8 | salmonella.sh,xs. or salmonella.ab,kf,ti. | 78529 |
| 9 | (Klebsiella or Proteus or Enterobacter or Morganella or Shigella or Citrobacter or Serratia or Yersinia or Providencia).sh,xs,ab,kf,ti | 86010 |
| 10 | exp *Enterobacteriaceae/ | 229772 |
| 11 | or/7-10 | 513658 |
| 12 | and/5-6,11 | 1566 |

**SEARCHED November 1, 2019**

**AGRICOLA**1970 to October 2019

| **#** | **Searches** | **Results** |
| --- | --- | --- |
| 1 | antibiotic resistance/ or multiple drug resistance/ | 10718 |
| 2 | (resistan* or antimicrobial* or microbial* or antibiotic* or anti biotic* or antibacterial* or multidrug* or multi drug* or extensively drug or multiple drug* or multiclass* or multi class* or multiple class* or extended-spectrum or CTX-M or TEM or SHV).tw,hw. | 450552 |
| 3 | (ESBL or "b-lactam*" or "beta-lactam*" or penicillin* or carbapenem* or cephalosporin* or moxalactam* or latamoxef*).tw,hw. | 7640 |
| 4 | or/1-3 [AMR] | 452731 |
| 5 | (fish or aquaculture or aquafarm* or fishfarm* or aquarium* or seafood or shellfish or crustacean* or shrimp* or prawn* or salmon or salmonid or salmonids).tw,hw. | 206097 |
| 6 | exp escherichia coli/ or (escherichia coli or "e coli" or ecoli).tw,hw. | 69734 |
| 7 | exp salmonella/ or salmonella.tw,hw. | 23365 |
| 8 | (Klebsiella or Proteus or Enterobacter or Morganella or Shigella or Citrobacter or Serratia or Yersinia or Providencia).tw,hw. | 14537 |
| 9 | exp Enterobacteriaceae/ | 60059 |
| 10 | or/6-9 | 102730 |
| 11 | and/4-5,10 | 1622 |

**SEARCHED November 1, 2019**

**Embase**1974 to 2019 October 31

| **#** | **Searches** | **Results** |
| --- | --- | --- |
| 1 | exp *antibiotic resistance/ or *multidrug resistance/ or *cross resistance/ | 71327 |
| 2 | (resistan* or antimicrobial* or microbial* or antibiotic* or anti biotic* or antibacterial* or multidrug* or multi drug* or extensively drug or multiple drug* or multiclass* or multi class* or multiple class* or extended-spectrum or CTX-M or TEM or SHV).ti,ab,kw. | 1878594 |
| 3 | exp *beta-lactams/ | 1674 |
| 4 | (ESBL or "B-lactam*" or "beta-lactam*" or penicillin* or carbapenem* or cephalosporin* or moxalactam* or latamoxef*).ti,ab,kw. | 124513 |
| 5 | or/1-4[AMR] | 1915820 |
| 6 | (fish or aquaculture or aquafarm* or fishfarm* or aquarium* or seafood or shellfish or crustacean* or shrimp* or prawn* or salmon or salmonid or salmonids).ti,ab,kw. | 244687 |
| 7 | exp *Escherichia coli/ or (Escherichia coli or "e coli" or ecoli).ti,ab,kw. | 350280 |
| 8 | exp *salmonella/ or salmonella.ti,ab,kw. | 76110 |
| 9 | (Enterobacteriaceae or Klebsiella or Proteus or Enterobacter or Morganella or Shigella or Citrobacter or Serratia or Yersinia or Providencia).ti,ab,kw. | 105262 |
| 10 | or/7-9 | 473897 |
| 11 | and/5-6,10 | 1815 |

**SEARCHED November 1, 2019**

**CAB Abstracts**

Database Field Guide CAB Abstracts 1973 to 2019 Week 43

| **#** | **Searches** | **Results** |
| --- | --- | --- |
| 1 | exp Drug Resistance/ | 59654 |
| 2 | (resistan* or antimicrobial* or microbial* or antibiotic* or anti biotic* or antibacterial* or multidrug* or multi drug* or extensively drug or multiple drug* or multiclass* or multi class* or multiple class* or extended-spectrum or CTX-M or TEM or SHV).ti,ab. | 812580 |
| 3 | exp beta-lactam antibiotics/ | 36684 |
| 4 | (ESBL or "B-lactam*" or "beta-lactam*" or penicillin* or carbapenem* or cephalosporin* or moxalactam* or latamoxef*).ti,ab. | 20791 |
| 5 | or/1-4 | 826556 |
| 6 | (fish or aquaculture or aquafarm* or fishfarm* or aquarium* or seafood or shellfish or crustacean* or shrimp* or prawn* or salmon or salmonid or salmonids).ti,ab. | 256129 |
| 7 | (Escherichia coli or e coli).ti,ab. | 112348 |
| 8 | salmonella.ti,ab. | 49922 |
| 9 | (Enterobacteriaceae or Klebsiella or Proteus or Enterobacter or Morganella or Shigella or Citrobacter or Serratia or Yersinia or Providencia).ti,ab. | 43237 |
| 10 | or/7-9 | 170981 |
| 11 | and/5-6,10 | 2313 |

**SEARCHED November 1, 2019**

**Food Science and Technology Abstracts** 1969 to 2019 October Week 5

| **#** | **Searches** | **Results** |
| --- | --- | --- |
| 1 | Antibiotics resistance/ | 6383 |
| 2 | (resistan* or antimicrobial* or microbial* or antibiotic* or anti biotic* or antibacterial* or multidrug* or multi drug* or extensively drug or multiple drug* or multiclass* or multi class* or multiple class* or extended-spectrum or CTX-M or TEM or SHV).ti,hw,ab. | 135044 |
| 3 | beta-lactam antibiotics/ or beta-lactamases/ | 454 |
| 4 | (ESBL or "B-lactam*" or "beta-lactam*" or penicillin* or carbapenem* or cephalosporin* or moxalactam* or latamoxef*).ti,hw,ab. | 3910 |
| 5 | or/1-4 | 135656 |
| 6 | (fish or aquaculture or aquafarm* or fishfarm* or aquarium* or seafood or shellfish or crustacean* or shrimp* or prawn* or salmon or salmonid or salmonids).ti,hw,ab. | 76998 |
| 7 | (Escherichia coli or e coli).ti,hw,ab. | 37710 |
| 8 | salmonella.ti,hw,ab. | 26080 |
| 9 | (Enterobacteriaceae or Klebsiella or Proteus or Enterobacter or Morganella or Shigella or Citrobacter or Serratia or Yersinia or Providencia).ti,hw,ab. | 15008 |
| 10 | or/7-9 | 63213 |
| 12 | and/5-6,10 | 1129 |

## Appendix 4: Search Strategy Updated February 11, 2021

This will include all the search string details and number of hits

**MEDLINE: SEARCHED February 11, 2021 by LS**

Database Field Guide Ovid MEDLINE(R) and Epub Ahead of Print, In-Process & Other Non-Indexed Citations, Daily and Versions(R) 1946 to February 10, 2021

| **#** | **Searches** | **Results** |
| --- | --- | --- |
| 1 | exp *Drug Resistance, Microbial/ or exp *Drug Resistance, Multiple/ | 78565 |
| 2 | (resistan* or antimicrobial* or microbial* or antibiotic* or anti biotic* or antibacterial* or multidrug* or multi drug* or extensively drug or multiple drug* or multiclass* or multi class* or multiple class* or extended-spectrum or CTX-M or TEM or SHV).ti,ab,kw,kf | 1645344 |
| 3 | exp *beta-Lactams/ | 82183 |
| 4 | (ESBL or "B-lactam*" or "beta-lactam*" or penicillin* or carbapenem* or cephalosporin* or moxalactam* or latamoxef*).ti,ab,kw,kf. | 120681 |
| 5 | or/1-4 [AMR] | 1711949 |
| 6 | (fish or aquaculture or aquafarm* or fishfarm* or aquarium* or seafood or shellfish or crustacean* or shrimp* or prawn* or salmon or salmonid or salmonids).ti,ab,kw,kf. | 219827 |
| 7 | Escherichia coli.sh,xs. or Escherichia coli.ab,kf,ti. or e coli.ab,kf,ti. | 402691 |
| 8 | salmonella.sh,xs. or salmonella.ab,kf,ti. | 82485 |
| 9 | (Klebsiella or Proteus or Enterobacter or Morganella or Shigella or Citrobacter or Serratia or Yersinia or Providencia).sh,xs,ab,kf,ti | 92176 |
| 10 | exp *Enterobacteriaceae/ | 237358 |
| 11 | or/7-10 | 539466 |
| 12 | and/5-6,11 | 1793 |
| 13 | limit 12 to yr="2019 -Current" | 354 |

**SEARCHED February 25, 2021 by LS**

**AGRICOLA**1970 to February 2021

| **#** | **Searches** | **Results** |
| --- | --- | --- |
| 1 | antibiotic resistance/ or multiple drug resistance/ | 20057 |
| 2 | (resistan* or antimicrobial* or microbial* or antibiotic* or anti biotic* or antibacterial* or multidrug* or multi drug* or extensively drug or multiple drug* or multiclass* or multi class* or multiple class* or extended-spectrum or CTX-M or TEM or SHV).tw,hw. | 493245 |
| 3 | (ESBL or "b-lactam*" or "beta-lactam*" or penicillin* or carbapenem* or cephalosporin* or moxalactam* or latamoxef*).tw,hw. | 8304 |
| 4 | 1 or 2 or 3 or AMR.af. | 495721 |
| 5 | (fish or aquaculture or aquafarm* or fishfarm* or aquarium* or seafood or shellfish or crustacean* or shrimp* or prawn* or salmon or salmonid or salmonids).tw,hw. | 218738 |
| 6 | exp escherichia coli/ or (escherichia coli or "e coli" or ecoli).tw,hw. | 74846 |
| 7 | exp salmonella/ or salmonella.tw,hw. | 24884 |
| 8 | (Klebsiella or Proteus or Enterobacter or Morganella or Shigella or Citrobacter or Serratia or Yersinia or Providencia).tw,hw. | 15815 |
| 9 | exp Enterobacteriaceae/ | 67908 |
| 10 | 6 or 7 or 8 or 9 | 110415 |
| 11 | 4 and 5 and 10 | 1802 |
| 12 | 11 and 2019:2021.(sa_year). | 252 |

**SEARCHED February 25, 2021 by KY**

**Embase**1974 to current

| **#** | **Searches** | **Results** |
| --- | --- | --- |
| 1 | exp *antibiotic resistance/ or *multidrug resistance/ or *cross resistance/ | 79621 |
| 2 | (resistan* or antimicrobial* or microbial* or antibiotic* or anti biotic* or antibacterial* or multidrug* or multi drug* or extensively drug or multiple drug* or multiclass* or multi class* or multiple class* or extended-spectrum or CTX-M or TEM or SHV).ti,ab,kw. | 2071669 |
| 3 | exp *beta-lactams/ | 1904 |
| 4 | (ESBL or "B-lactam*" or "beta-lactam*" or penicillin* or carbapenem* or cephalosporin* or moxalactam* or latamoxef*).ti,ab,kw. | 134677 |
| 5 | or/1-4[AMR] | 2110302 |
| 6 | (fish or aquaculture or aquafarm* or fishfarm* or aquarium* or seafood or shellfish or crustacean* or shrimp* or prawn* or salmon or salmonid or salmonids).ti,ab,kw. | 266659 |
| 7 | exp *Escherichia coli/ or (Escherichia coli or "e coli" or ecoli).ti,ab,kw. | 371263 |
| 8 | exp *salmonella/ or salmonella.ti,ab,kw. | 80838 |
| 9 | (Enterobacteriaceae or Klebsiella or Proteus or Enterobacter or Morganella or Shigella or Citrobacter or Serratia or Yersinia or Providencia).ti,ab,kw. | 115361 |
| 10 | or/7-9 | 504746 |
| 11 | and/5-6,10 | 2065 |
| 12 | Limit to 2019-present | 401 |

**SEARCHED February 25, 2021 by KY**

**CAB Abstracts**

**CAB Abstracts** 1973 to 2021 Week 07

| **#** | **Searches** | **Results** |
| --- | --- | --- |
| 1 | exp Drug Resistance/ | 65890 |
| 2 | (resistan* or antimicrobial* or microbial* or antibiotic* or anti biotic* or antibacterial* or multidrug* or multi drug* or extensively drug or multiple drug* or multiclass* or multi class* or multiple class* or extended-spectrum or CTX-M or TEM or SHV).ti,ab. | 878477 |
| 3 | exp beta-lactam antibiotics/ | 39849 |
| 4 | (ESBL or "B-lactam*" or "beta-lactam*" or penicillin* or carbapenem* or cephalosporin* or moxalactam* or latamoxef*).ti,ab. | 22721 |
| 5 | or/1-4 | 892936 |
| 6 | (fish or aquaculture or aquafarm* or fishfarm* or aquarium* or seafood or shellfish or crustacean* or shrimp* or prawn* or salmon or salmonid or salmonids).ti,ab. | 276719 |
| 7 | (Escherichia coli or e coli).ti,ab. | 120220 |
| 8 | salmonella.ti,ab. | 53110 |
| 9 | (Enterobacteriaceae or Klebsiella or Proteus or Enterobacter or Morganella or Shigella or Citrobacter or Serratia or Yersinia or Providencia).ti,ab. | 46850 |
| 10 | or/7-9 | 183057 |
| 11 | and/5-6,10 | 2598 |
| 12 | Limit to 2019-current | 376 |

**SEARCHED February 25, 2021 by LS**

**Food Science and Technology Abstracts** 1969 to 2021 February Week 3

| **#** | **Searches** | **Results** |
| --- | --- | --- |
| 1 | Antibiotics resistance/ | 7035 |
| 2 | (resistan* or antimicrobial* or microbial* or antibiotic* or anti biotic* or antibacterial* or multidrug* or multi drug* or extensively drug or multiple drug* or multiclass* or multi class* or multiple class* or extended-spectrum or CTX-M or TEM or SHV).ti,hw,ab. | 150510 |
| 3 | beta-lactam antibiotics/ or beta-lactamases/ | 504 |
| 4 | (ESBL or "B-lactam*" or "beta-lactam*" or penicillin* or carbapenem* or cephalosporin* or moxalactam* or latamoxef*).ti,hw,ab. | 4314 |
| 5 | or/1-4 | 151157 |
| 6 | (fish or aquaculture or aquafarm* or fishfarm* or aquarium* or seafood or shellfish or crustacean* or shrimp* or prawn* or salmon or salmonid or salmonids).ti,hw,ab. | 82600 |
| 7 | (Escherichia coli or e coli).ti,hw,ab. | 40361 |
| 8 | salmonella.ti,hw,ab. | 27950 |
| 9 | (Enterobacteriaceae or Klebsiella or Proteus or Enterobacter or Morganella or Shigella or Citrobacter or Serratia or Yersinia or Providencia).ti,hw,ab. | 15778 |
| 10 | or/7-9 | 67558 |
| 11 | and/5-6,10 | 1244 |
| 12 | 11 and 2019:2021.(sa_year). | 211 |

**TOTAL Search 1 = 8445**

**Total Search 2 = 1594**

**Google scholar + hand searching of reference lists = 6**

**De-duplication = 5132 removed**

**References screened = 4913**

# S2. Excluded studies that investigated the presence of ESBL-E in shrimp and salmon and found none (n=6).

| Study | Country study was conducted | Origin of seafood species (year samples were collected) | Seafood commodities investigated | Enterobacteriaceae isolated and investigated |
| --- | --- | --- | --- | --- |
| Ahmed et al., 2015 | Japan | Chile & Indonesia (2006) | **Salmon**  **Shrimp**  Alfonsin, *Pagrus major*, Shijimi | *E. cloacae*  *C. freundii*  *Pantoea* spp. |
| Janecko et al., 2016 | Canada | Vietnam & Bangladesh (2011-2015) | **Shrimp**  Bivalve mollusks, Cephalopods | *E. cloaceae*  *E. aerogenes* |
| Kakatkar et al., 2011 | India | India (2006-2008) | **Shrimp**  Anchovy, Bombay duck, Butter catfish, Catla, Mangur, Rohu | *Salmonella* Ohio  *S.* Oslo  S. Tennessee  *S.* Typhimurium  *S.* Weltevreden |
| Li et al., 2018 | China | China (2015-2016) | **Shrimp** | *S.* Weltevreden |
| Nguyen et al., 2016a | Vietnam | Vietnam (2012-2015) | **Shrimp** | *Salmonella* spp.^a^ |
| Beshiru et al., 2019 | Nigeria | Nigeria | Shrimp | *S.* Enteritidis  S. Typhimurium  *Salmonella* spp.  Note: this study found the presence of *bla*_TEM_ gene but the isolates did not show resistance to third generation cephalosporins tested. The study did not formally investigate ESBL’s phenotypically. So the decision was made to exclude this study. |

^a^ Braenderup, Chester, Bareill, Kentucky, Give, Virchow, Agona, Anatum, Corvallis, Enteritidis, Lexington, Newport, Saintpaul, Stanley, Tennessee, Thompson, Typhimurium, Urbana, Wandsworth, Weltevreden.

# S3. Excluded studies that did not report phenotypic/genotypic ESBL detection separately for each commodity (i.e., unextractable) (n=10).

| **Study** | Country study was conducted | Origin of seafood species (year samples were collected) | Seafood commodities investigated | Enterobacteriaceae isolated and investigated |
| --- | --- | --- | --- | --- |
| Guo et al., 2016 | China | China | Fresh/raw seafood Shrimp | *Klebsiella pneumoniae* |
| Hussein et al., 2018 | Egypt | Egypt | Sandwich Shrimp | E.coli |
| Neelawan et al., 2018 | Thailand | Thailand | Fresh/raw seafood Shrimp/Prawn | Enterobacteriaceae |
| Nguyen et al., 2016b | Vietnam | Vietnam | Fish/**Shrimp** | E.coli |
| Roschanski et al., 2017 | Germany | Not provided | Seafood Shrimp | Enterobacteriaceae |
| Sivaraman et al., 2017 | India | India | Seafood Prawn | E.coli |
| Veldman et al., 2020 | Netherlands | Netherlands | Fish and **Shrimp** | E. coli |
| Yamaguchi et al., 2018 | Vietnam | Vietnam | Fish and **Shrimp** | *E.coli* |
| Ye et al., 2013 | China | China | Fish and **Shrimp** | Enterobacteriaceae |
| Cheong et al., 2014 | Malaysia | Malaysia | Sushi  **Salmon** and **Shrimp** | Enterobacteriaceae |

**References**

**Ahmed AM, et al.** (2015). Seafood as a reservoir of Gram-negative bacteria carrying integrons and antimicrobial resistance genes in Japan. Biomedical and Environmental Sciences, **28**, 924-927.

**Beshiru A, Igbinosa IH, Igbinosa EO.** (2019). Prevalence of Antimicrobial Resistance and Virulence Gene Elements of Salmonella Serovars From Ready-to-Eat (RTE) Shrimps. Frontiers in microbiology, **10**, 1613.

**Cheong H, et al.** (2014). β-lactamase gene blaSHV detected in bacteria isolated from retail sushi in Kampar, Malaysia. Biomedical Research, **25**: 25-31.

**Guo Y, et al.** (2016). Frequency, Antimicrobial Resistance and Genetic Diversity of Klebsiella pneumoniae in Food Samples. PloS one, **11**, e0153561.

**Hussein MA, et al.** (2018). Virulence and antimicrobial resistance genes of Escherichia coli in ready to eat sandwiches in Sharkia governorate. Slovenian Veterinary Research, **55**, 383-392.

**Janecko N, et al.** (2016). Carbapenem-Resistant Enterobacter spp. in Retail Seafood Imported from Southeast Asia to Canada. Emerging infectious diseases, **22**, 1675-1677.

**Kakatkar AS, et al.** (2011). Molecular characterization of antibiotic resistant Salmonella isolates from Indian foods. Food research international., **44**, 3272-3275.

**Li B, et al.** (2018). Whole genome sequencing analysis of Salmonella enterica serovar Weltevreden isolated from human stool and contaminated food samples collected from the Southern coastal area of China. International journal of food microbiology, **266**, 317-323.

**Neelawan P, Pongrawee N.** (2018). Diversity and antibiotic resistance patterns of enterobacteria isolated from seafood in Thailand. CyTA -- Journal of Food, **16**, 793-800.

**Nguyen DTA, et al.** (2016a). Prevalence, antibiotic resistance, and extended-spectrum and AmpC beta-lactamase productivity of Salmonella isolates from raw meat and seafood samples in Ho Chi Minh City, Vietnam. International journal of food microbiology, **236**, 115-122.

**Nguyen DP, et al.** (2016b). Dissemination of Extended-Spectrum beta-Lactamase- and AmpC beta-Lactamase-Producing Escherichia coli within the Food Distribution System of Ho Chi Minh City, Vietnam. BioMed research international, **2016**, 8182096.

**Roschanski N, et al.** (2017). VIM-1 carbapenemase-producing Escherichia coli isolated from retail seafood, Germany 2016. Euro surveillance : bulletin Europeen sur les maladies transmissibles = European communicable disease bulletin, **22**.

**Sivaraman GK, et al.** (2017). Prevalence of extended-spectrum beta -lactamase producing Escherichia coli in seafood from the retail fishery outlets of Veraval, Gujarat, India. Journal of Environmental Biology, **38**, 523-526.

**Veldman KT, et al.** (2020). MARAN 2020. Monitoring of Antimicrobial Resistance and Antibiotic Usage in Animals in the Netherlands in 2019, 77-pp.

**Yamaguchi T, et al.** (2018). The presence of colistin resistance gene mcr-1 and -3 in ESBL producing Escherichia coli isolated from food in Ho Chi Minh City, Vietnam. FEMS microbiology letters, **365**.

**Ye L, et al.** (2013). Antibiotic-resistant bacteria associated with retail aquaculture products from Guangzhou, China. Journal of food protection, **76**, 295-301.
